# Impact of visual impairments on cognitive functions in older adults: insights from NHANES data

**DOI:** 10.3389/fpubh.2024.1455550

**Published:** 2024-11-18

**Authors:** Xiangxiang Fu, Zhenni Du, Jianing Ying, Qianwei Zhu

**Affiliations:** ^1^Yuyao Maternity and Child Health Care Hospital (Yuyao Second People's Hospital), Ningbo, China; ^2^Ningbo Eye Hospital, Ningbo, China

**Keywords:** visual impairment, cognitive decline, older adults population, non-linear associations, NHANES

## Abstract

**Background:**

Visual impairments (VI) are common in the older adults and may affect cognitive functions through mechanisms such as reduced sensory input and increased social isolation. Although current understanding of this association is incomplete, evidence suggests a potential link between poor vision and cognitive decline. This study aims to investigate the relationship between Subjective Visual Impairment (SVI), Objective Visual Impairment (OVI), and cognitive decline in the older adults, and assess whether these associations remain significant after controlling for multiple confounding factors.

**Methods:**

Data from the National Health and Nutrition Examination Survey (NHANES) for the years 1999–2002 were utilized, focusing on participants aged 60 and above. A total of 2,522 eligible participants were analyzed to assess their cognitive function and visual status. Weighted logistic regression models were used to explore the relationship between cognitive status and VI, progressively adjusting for confounding variables. Non-linear associations between cognitive score and VI were further explored using restricted cubic spline analysis.

**Results:**

Visual impairments were significantly associated with worse cognitive function. Participants with SVI had a 3.437-fold higher risk of cognitive decline compared to those without SVI (95% CI: 2.648–4.461, *p* < 0.001). After adjusting for multiple covariates, the association remained significant (adjusted *OR* for SVI: 1.921, 95% CI: 1.346–2.742, *p* = 0.001; adjusted *OR* for OVI: 3.075, 95% CI: 1.740–5.433, *p* = 0.001). The restricted cubic spline analysis revealed a non-linear relationship between cognitive score and visual impairment, suggesting that the impact of visual impairment on cognitive decline varies across different levels of cognitive function.

**Conclusion:**

This study highlights a significant association between visual impairment and cognitive decline, even after controlling for multiple potential influencers. The findings support the integration of vision assessments into older adults care to identify and address vision problems early, potentially mitigating cognitive decline. The discovery of non-linear relationships further suggests that vision interventions may be particularly vital at certain stages of cognitive scores.

## Introduction

Cognitive function is crucial for maintaining independence, quality of life, and overall well-being in older adults. As people age, cognitive decline can lead to difficulties in performing daily activities, increased dependence on caregivers, and a higher risk of institutionalization (1). Cognitive impairment, including conditions such as mild cognitive impairment and dementia, affects a significant proportion of the older adults population (2). The growing prevalence of cognitive impairment poses major challenges for healthcare systems and necessitates effective strategies for prevention and management (3).

Vision plays a vital role in maintaining daily activities and overall well-being (4). Good vision is essential for engaging in daily tasks, maintaining mobility, and ensuring safety (5). Visual impairment (VI) refers to a range of conditions that reduce an individual’s ability to see clearly, even with corrective measures such as glasses or contact lenses. VI is typically assessed in two ways: subjective visual impairment (SVI), based on an individual’s self-reported vision, and objective visual impairment (OVI), measured through clinical tests like visual acuity. While subjective and objective measures offer valuable insights into the link between vision and cognitive decline, they may capture different aspects of visual health. VI is common among older adults, with conditions such as age-related macular degeneration, cataracts, and glaucoma being prevalent and leading causes of disability. Impaired vision can severely limit an individual’s ability to navigate their environment and perform routine tasks, resulting in reduced physical activity, social isolation, and a higher risk of falls and accidents (6). These consequences significantly impact the quality of life for the older adults (7).

Existing research suggests that there may be a link between VI and cognitive decline, though the relationship is not yet fully understood (8). For instance, a Canadian Longitudinal Study showed that no significant association between visual impairment (such as age-related macular degeneration and cataract) and changes in cognitive test scores over 3 years. However, patients with glaucoma exhibited faster declines in processing speed during the tests (9). Poor vision may contribute to cognitive impairment through various mechanisms, such as reduced sensory input and increased social isolation (10, 11). Another study explored the predictive role of auditory and visual function on cognitive performance, finding that hearing impairment had a greater impact on executive function in older adults and individuals with more health conditions, while visual impairment had a stronger effect on executive function in those without a high school education. The study also suggested that future research should incorporate both self-reported and objective measures to better understand the relationship between sensory function and cognitive health (12). A comprehensive study by Hämäläinen et al. (13) emphasized that self-reported sensory impairments, including vision, often underreport objective deficits, highlighting the need for more precise behavioral measures to accurately assess sensory decline. This underscores the importance of incorporating both self-reported and objective measures in future research to gain a clearer understanding of sensory impairments and their broader impact on daily functioning and social participation (13). Despite the potential link, substantial population studies are needed to elucidate the extent and nature of this association. This study hypothesizes that subjective visual impairment (SVI) and objective visual impairment (OVI) is associated with cognitive decline in older adults, warranting further investigation.

The National Health and Nutrition Examination Survey (NHANES) provides an ideal dataset for investigating this relationship. NHANES is a continuous program that assesses the health and nutritional status of adults and children in the United States through interviews and physical examinations. The 1999–2002 NHANES cycle includes detailed information on both cognitive function and vision status, making it particularly suitable for this study. The use of NHANES data ensures that the findings are representative of the U.S. population, enhancing the generalizability of the study results.

By leveraging the comprehensive NHANES dataset, this study aims to thoroughly investigate the relationship between VI and cognitive decline in the older adults population. Understanding this relationship is crucial for developing targeted interventions to mitigate cognitive decline in individuals with visual impairments, ultimately improving the quality of life for older adults.

## Methods

### Study population

The present study utilized data from the National Health and Nutrition Examination Survey (NHANES) for the years 1999–2002. NHANES is a program designed to assess the health and nutritional status of adults and children in the United States, uniquely combining interviews and physical examinations. The data collected by NHANES provide a comprehensive overview of the health status and behaviors of the U.S. population, making it an invaluable resource for public health research.

Participants included in this analysis were restricted to those aged 60 years and older. Initially, individuals younger than 60 years were excluded from the dataset. Subsequently, participants with missing vision and cognitive data were excluded. This rigorous exclusion process ensured that the study focused on a specific and relevant population, allowing for a more accurate investigation into the relationship between VI and cognitive decline. It is important to note that we specifically chose data from the 1999–2002 NHANES cycle because the surveys in these years included both cognitive function and visual data. This unique combination of data types was essential for our analysis, enabling a comprehensive exploration of the association between VI and cognitive decline.

**Figure 1:**
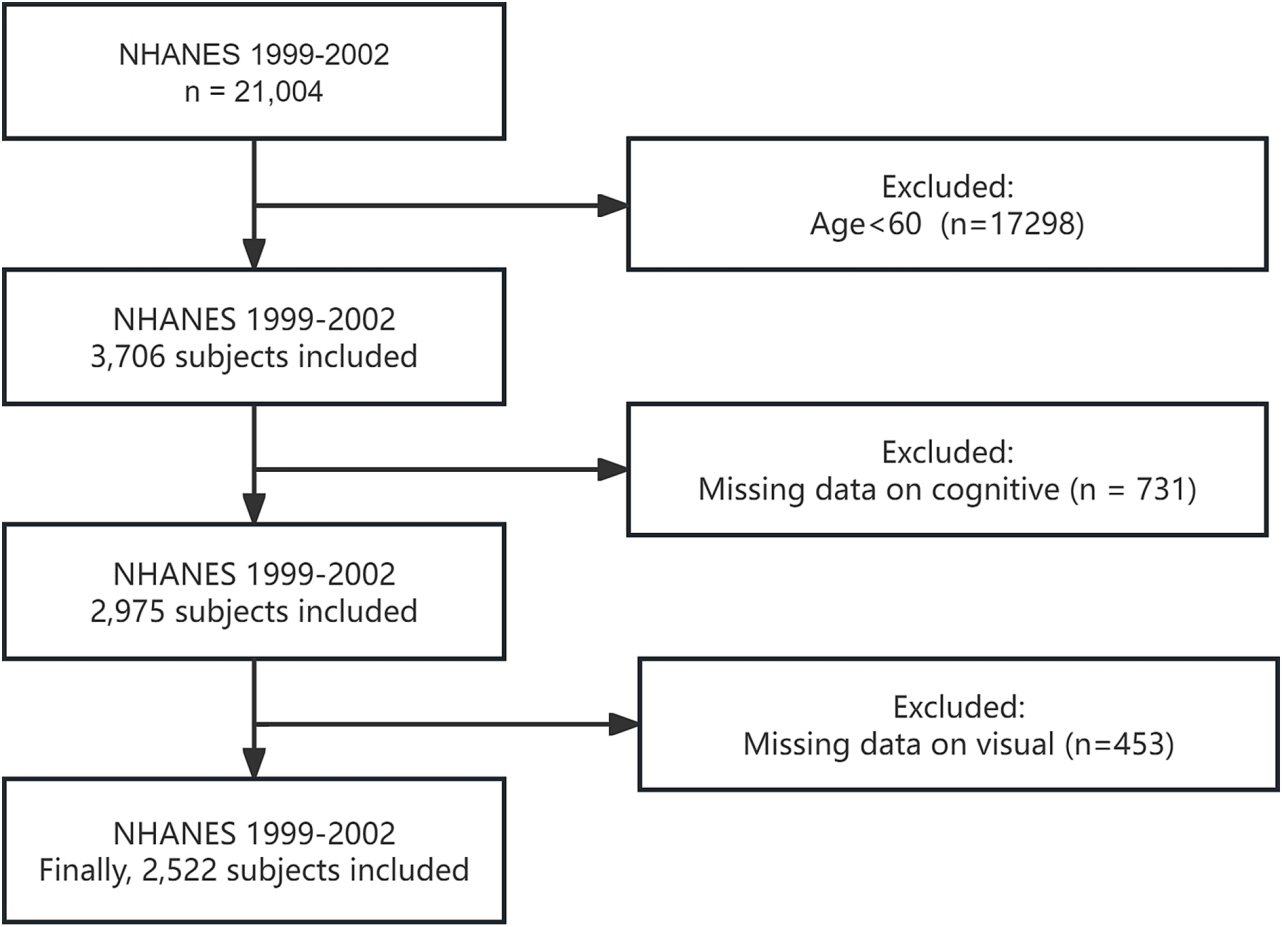
Screening conditions and process for the study population.

After applying these exclusion criteria, a total of 2,522 participants were included in the final analysis. The detailed participant selection process is illustrated in Figure 1, which provides a clear flowchart of the inclusion and exclusion criteria applied to reach the final study population.

### Assessment of cognition and vision

#### Cognition

Cognitive function was assessed using the CFDRIGHT – Score: number correct variable from the CFQ module in the NHANES database. This variable measures the total score obtained by respondents based on their answers to a series of questions designed to evaluate cognitive function. The score ranges from 0 to 133, with higher scores indicating stronger cognitive function. This scoring system provides a comprehensive assessment of respondents’ cognitive abilities based on their performance on the administered tasks.

#### Vision

In this study, both subjective self-reported vision status and objective visual acuity tests were used to evaluate eyesight. Subjective Visual Impairment (SVI) was determined based on participants’ responses to the question regarding their general vision status: “Would you say your vision, with glasses or contact lenses if you wear them, is…”. Participants were classified as non-SVI if their response was “excellent” or “good.” Conversely, if their response was “fair,” “poor,” or “very poor,” they were classified as SVI. Any data coded as “do not know,” “refused,” or “missing” were excluded from the analysis. Objective visual impairment (OVI) was identified based on the results of visual acuity (VA) tests, using the participant’s usual corrective lenses, if applicable. Participants with a VA of 20/40 or better in at least one eye were categorized as non-OVI, while those with a VA worse than 20/40 in the better eye were classified as having OVI (14).

### Covariates

Covariates included age, gender, race, education level, family income-to-poverty ratio (PIR), smoking status, alcohol consumption, hypertension, and stroke. Race: Race was categorized based on NHANES-defined racial/ethnic groups. Education Level: Education level was classified according to the highest degree of education completed by the participant. PIR: The PIR was used to measure the general economic status of the household. Smoking Status: Smoking status was determined by asking participants whether they had smoked more than 100 cigarettes in their lifetime and whether they currently smoke. Participants who answered “yes” to both questions were classified as current smokers. Alcohol Consumption: Alcohol consumption was assessed based on participants’ self-report of drinking at least 12 alcoholic beverages in the past year. Participants who met this criterion were classified as alcohol consumers. Definitions of hypertension, diabetes, and stroke were assessed by asking: “Have you ever been told by a doctor or other health professional that you have hypertension/diabetes/stroke?” These covariates were included in the analysis to control for potential confounding factors that could influence the relationship between VI and cognitive decline.

### Statistical analysis

Given that NHANES employs a complex, multistage probability sampling design, we used weighted procedures to ensure the national representativeness of the U.S. population. According to the NHANES analytic guidelines,^1^ analyses were conducted using raw data and weighted estimates, with the weights for each cycle being directly available from the database.

Categorical variables were described using frequencies (*n*) and percentages (%), and were analyzed using the chi-square test. All continuous data were non-normally distributed. Non-normally distributed continuous variables were described using medians (M) and interquartile ranges (Q1, Q3), and were analyzed using the Mann–Whitney *U* test. Cognitive scores were dichotomized into adequate cognition and limited cognition based on the median score. Weighted logistic regression analyses were conducted to explore the association between cognitive status and poor vision, with a total of four models constructed by progressively adjusting for covariates. Subgroup analyses and interaction analyses were also performed to further explore the stability of the relationship between cognitive status and poor vision. Forest plots were generated to visually present the results of the logistic regression analyses. Finally, we utilized restricted cubic spline analyses to explore the non-linear relationship between cognitive status and poor vision. Since vision is a categorical variable and cognitive score is a continuous variable, the cognitive score was included as the *x*-variable in the restricted cubic spline analysis. All data were collated and analyzed using R version 4.0.0. A *p-*value of less than 0.05 was considered statistically significant.

## Results

### Characteristics of the study participants

As shown in Table 1, the study included a total of 2,522 participants, of which 1,267 had adequate cognition and 1,255 had limited cognition. SVI was more prevalent in the limited cognition group (37.85%) compared to the adequate cognition group (15.47%) (*p* < 0.001). OVI was also significantly more common in the limited cognition group, with 16.02% of participants exhibiting OVI, compared to 4.89% in the adequate cognition group (*p* < 0.001). Age: the median age was higher in the limited cognition group (72 years) compared to the adequate cognition group (68 years) (*p* < 0.001). Gender: slightly more males were in the limited cognition group (52.91%) compared to the adequate cognition group (45.62%) (*p* < 0.001). Race and education: significant differences were observed, with non-Hispanic Whites and individuals with lower education levels being more prevalent in the limited cognition group (*p* < 0.001). Poverty income ratio: a lower median PIR was observed in the limited cognition group (*p* < 0.001). Alcohol use: alcohol use was higher in the adequate cognition group compared to the limited cognition group (*p* < 0.001). Hypertension, diabetes and stroke were more prevalent in the limited cognition group.

**Table 1:** Characteristics of study participants by cognitive status.

Variables	Total (*n* = 2,522)	Adequate cognition (*n* = 1,267)	Limited cognition (*n* = 1,255)	Statistic	*P*
SVI, *n*(%)				161.70	<0.001
No	1851 (73.39)	1,071 (84.53)	780 (62.15)		
Yes	671 (26.61)	196 (15.47)	475 (37.85)		
OVI, n(%)				83.50	<0.001
No	2,259 (89.57)	1,205 (95.11)	1,054 (83.98)		
Yes	263 (10.43)	62 (4.89)	201 (16.02)		
Age, M (Q₁, Q₃)	70.00 (64.00, 77.00)	68.00 (63.00, 75.00)	72.00 (66.00, 80.00)	−10.16	<0.001
Gender, *n*(%)				13.40	<0.001
Male	1,242 (49.25)	578 (45.62)	664 (52.91)		
Female	1,280 (50.75)	689 (54.38)	591 (47.09)		
Race, *n*(%)				214.53	<0.001
Non-Hispanic White	482 (19.11)	156 (12.31)	326 (25.98)		
Non-Hispanic Black	94 (3.73)	25 (1.97)	69 (5.50)		
Mexican American	1,533 (60.79)	942 (74.35)	591 (47.09)		
Other Race – Including Multi-Racia	360 (14.27)	112 (8.84)	248 (19.76)		
Other Hispanic	53 (2.10)	32 (2.53)	21 (1.67)		
Education, *n*(%)				566.22	<0.001
Less Than 9th Grade	551 (21.88)	69 (5.45)	482 (38.50)		
9–11th Grade	434 (17.24)	162 (12.80)	272 (21.73)		
High School Grad/GED or Equivalent	620 (24.62)	365 (28.83)	255 (20.37)		
Some College or AA degree	510 (20.25)	351 (27.73)	159 (12.70)		
College Graduate or above	403 (16.00)	319 (25.20)	84 (6.71)		
PIR, M (Q₁, Q₃)	2.22 (1.24, 4.00)	3.16 (1.90, 5.00)	1.55 (1.03, 2.55)	−19.73	<0.001
Smoke, *n*(%)				1.70	0.192
No	2,214 (87.79)	1,123 (88.63)	1,091 (86.93)		
Yes	308 (12.21)	144 (11.37)	164 (13.07)		
Alcohol use, *n*(%)				22.52	<0.001
No	970 (39.37)	431 (34.73)	539 (44.07)		
Yes	1,494 (60.63)	810 (65.27)	684 (55.93)		
Hypertension, *n*(%)				9.51	0.002
No	1,215 (48.33)	650 (51.38)	565 (45.24)		
Yes	1,299 (51.67)	615 (48.62)	684 (54.76)		
Diabetes, *n*(%)				36.91	<0.001
No	2041 (80.99)	1,086 (85.71)	955 (76.22)		
Yes	479 (19.01)	181 (14.29)	298 (23.78)		
Stroke, *n*(%)				44.30	<0.001
No	2,354 (93.60)	1,223 (96.83)	1,131 (90.34)		
Yes	161 (6.40)	40 (3.17)	121 (9.66)		

### Association between cognitive status and visual impairment

**Figure 2:**
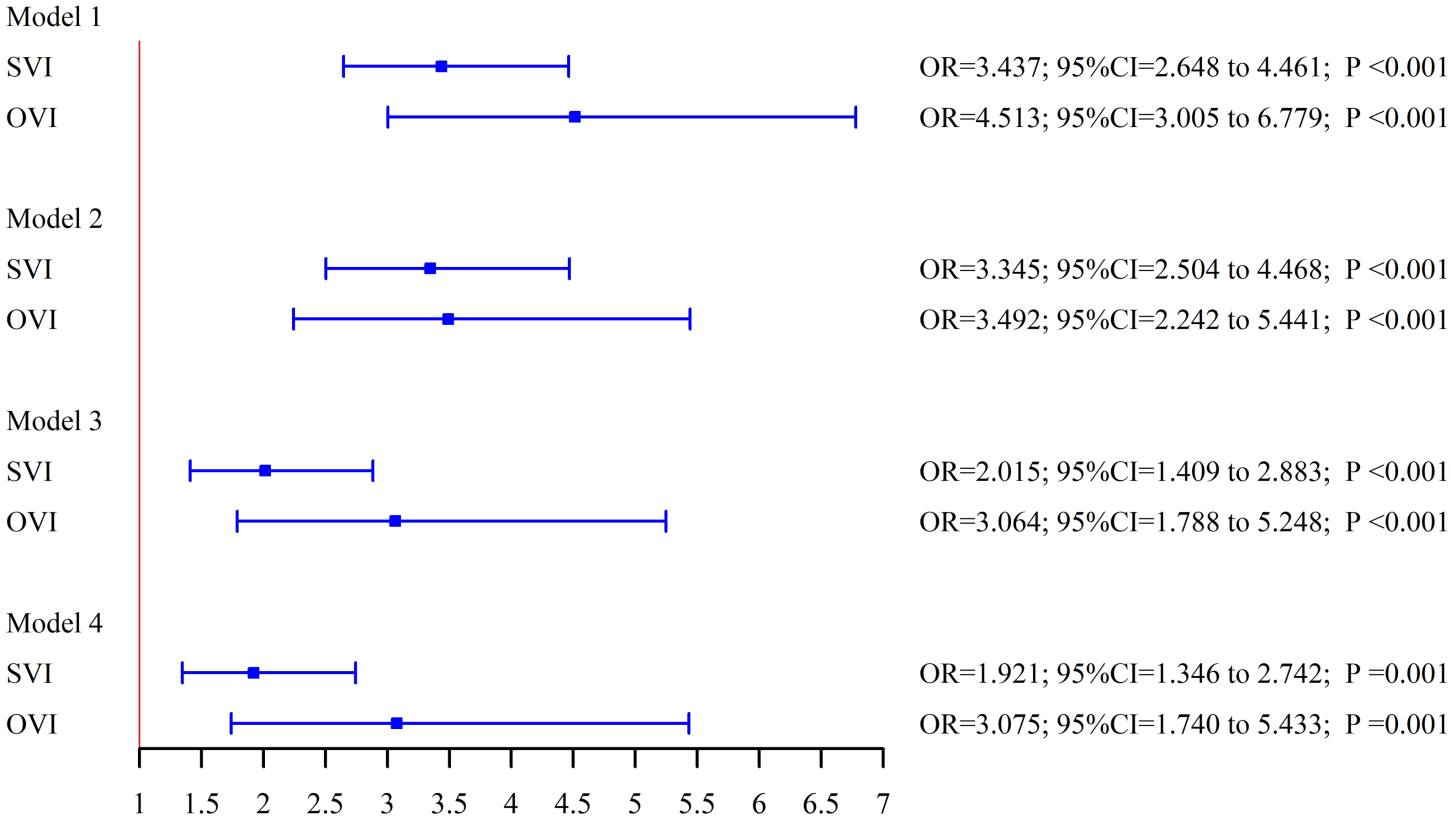
Weighted logistic regression forest plot.

As shown in Table 2 and Figure 2, the association between cognitive status and both SVI and OVI remained significant after progressively adjusting for covariates. Participants with either SVI or OVI consistently exhibited poorer cognitive function. In the unadjusted models (Model 1), the odds ratios (ORs) for limited cognition in those with SVI and OVI were significantly higher compared to participants without visual impairments. After adjusting for a range of variables, the OR for limited cognition in participants with SVI was 1.921 (95% CI: 1.346–2.742, *p* = 0.001), and for those with OVI, the OR was 3.075 (95% CI: 1.740–5.433, *p* = 0.001). These results indicate that both subjective and objective visual impairments are strongly associated with an increased risk of cognitive impairment, even after accounting for multiple potential confounding factors.

**Table 2:** Association between cognitive status and visual impairment.

	Model 1		Model 2		Model 3		Model 4	
	*OR* (95% CI)	*P*	*OR* (95% CI)	*P*	*OR* (95% CI)	*P*	*OR* (95% CI)	*P*
No-SVI	Reference		Reference		Reference		Reference	
SVI	3.437 (2.648, 4.461)	<0.001	3.345 (2.504, 4.468)	<0.001	2.015 (1.409, 2.883)	<0.001	1.921 (1.346, 2.742)	0.001
No-OVI	Reference		Reference		Reference		Reference	
OVI	4.513 (3.005, 6.779)	<0.001	3.492 (2.242, 5.441)	<0.001	3.064 (1.788, 5.248)	<0.001	3.075 (1.740, 5.433)	0.001

Model 1: no adjustment for any covariates; Model 2: controlled for age, gender and race; Model 3: additionally adjusted for education, and PIR on Model 2; Model 4: further adjusted for smoking/alcohol history, hypertension, diabetes, and stroke on Model 3; 95%CI, Confidence Interval.

### Subgroup and interaction analysis

Table 3 present the results of the subgroup and interaction analyses. Subgroup analysis showed that the association between cognitive status and SVI was generally stronger in males, while for OVI, the association was more pronounced in females. The age-related differences were consistent, with both SVI and OVI showing stronger associations in participants aged 70–85 years. No significant interactions were found between visual impairments and other variables, such as smoking, alcohol use, hypertension, and diabetes, indicating that the relationship between visual impairment and cognitive function remained consistent across these subgroups.

**Table 3:** Subgroup analysis and interaction analysis of the relationship between cognitive state and visual impairment.

Subgroup	*OR* (95% CI)	*P*	*P* for interaction
SVI			
Sex			0.601
Male	2.130 (1.347, 3.369)	0.003	
Female	1.783 (1.159, 2.741)	0.011	
Age strata			0.335
60–69	1.532 (0.863, 2.719)	0.136	
70–85	2.388 (1.612, 3.538)	<0.001	
Smoke			0.455
No	2.004 (1.382, 2.906)	0.001	
Yes	1.434 (0.584, 3.521)	0.409	
Alcohol use			0.869
No	1.847 (1.009, 3.382)	0.047	
Yes	2.013 (1.389, 2.917)	0.001	
Hypertension			0.504
No	1.676 (0.896, 3.137)	0.101	
Yes	2.119 (1.404, 3.198)	0.001	
Diabetes			0.099
No	2.147 (1.517, 3.04)	<0.001	
Yes	1.175 (0.584, 2.367)	0.634	
OVI			
Sex			0.118
Male	2.017 (0.815, 4.995)	0.122	
Female	4.114 (2.437, 6.944)	<0.001	
Age strata			0.966
60–69	3.482 (1.019, 11.899)	0.047	
70–85	3.371 (1.751, 6.490)	0.001	
Smoke			0.319
No	2.886 (1.547, 5.381)	0.002	
Yes	8.909 (2.136, 37.161)	0.005	
Alcohol use			0.729
No	2.768 (1.374, 5.580)	0.007	
Yes	3.378 (1.366, 8.355)	0.011	
Hypertension			0.236
No	5.099 (1.797, 14.471)	0.004	
Yes	2.486 (1.331, 4.643)	0.007	
Diabetes			0.773
No	3.210 (1.729, 5.956)	0.001	
Yes	2.656 (0.768, 9.186)	0.116	

### Non-linear associations

**Figure 3:**
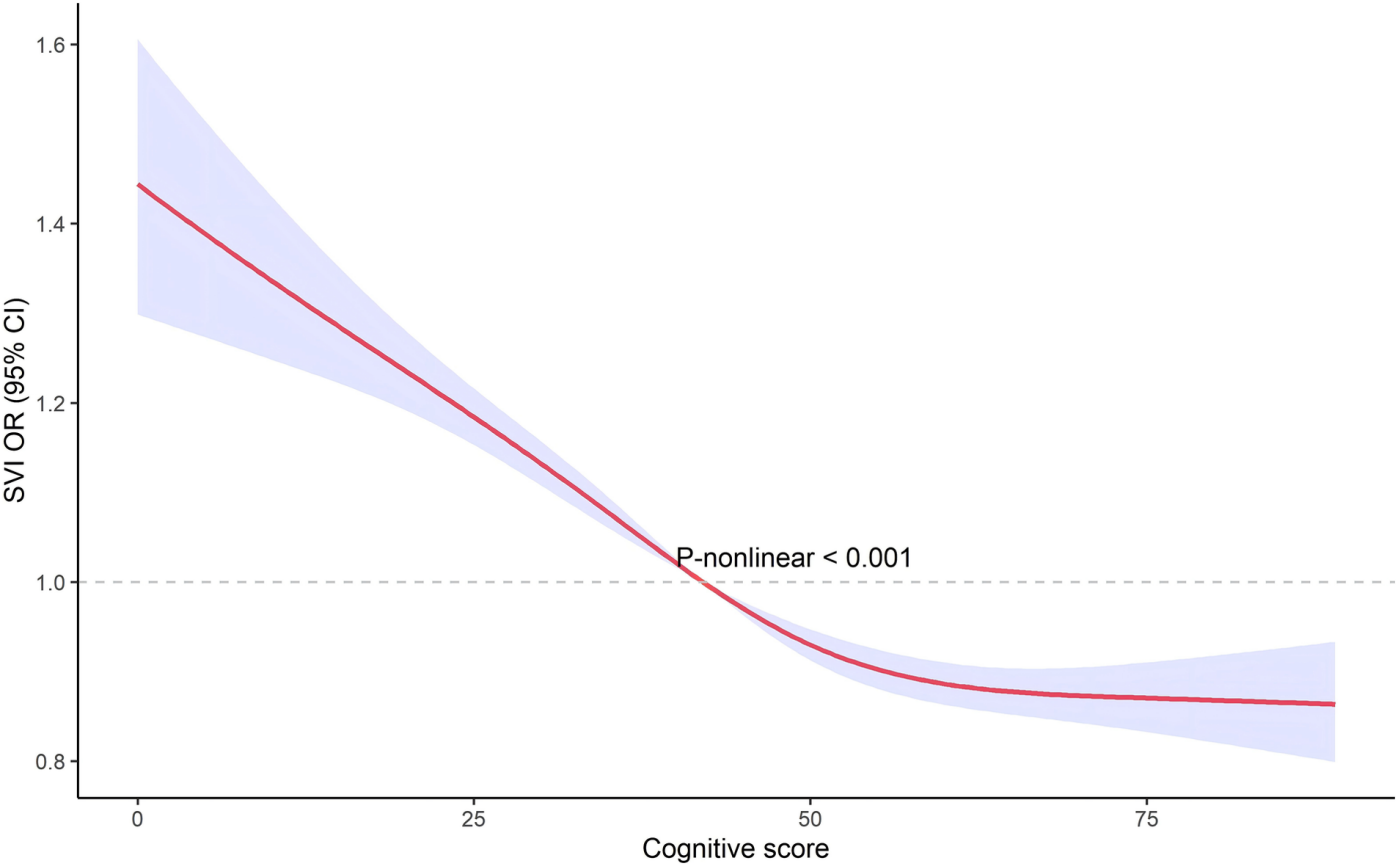
The non-linear associations between cognitive score and SVI by restricted cubic splines.

**Figure 4:**
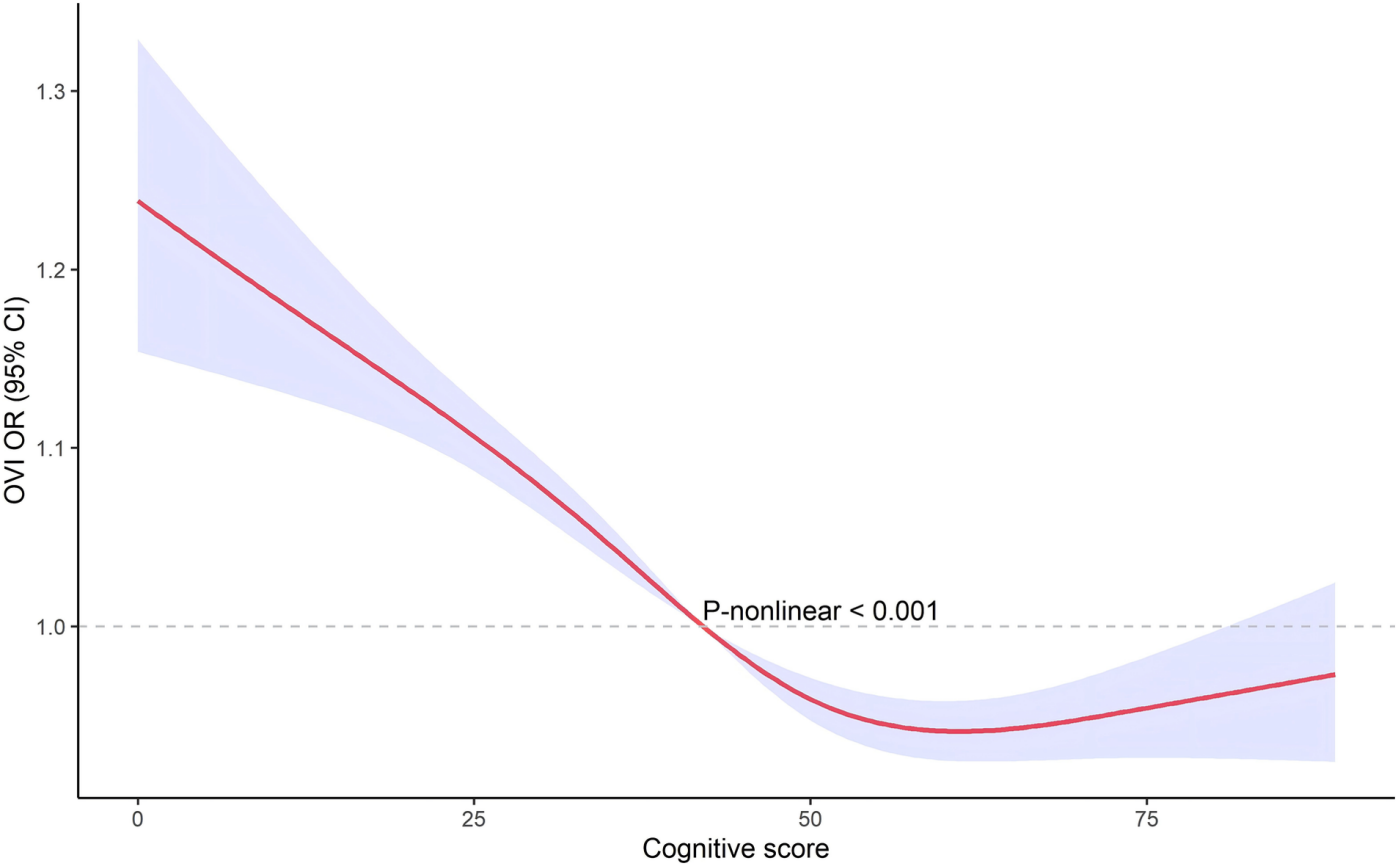
The non-linear associations between cognitive score and OVI by restricted cubic splines.

The restricted cubic spline analysis, illustrated in Figures 3, 4, revealed a non-linear relationship between cognitive score and visual impairment. This non-linear association suggests that the relationship between cognitive function and visual impairment is not uniform across all levels of cognitive scores. Specifically, the impact of poor vision on cognitive function may vary depending on the cognitive score range. This non-linearity indicates that there may be thresholds or specific ranges of cognitive scores where the effect of visual impairment is more pronounced.

## Discussion

### Significant association between VI and cognitive decline

This study demonstrated a significant association between both SVI and OVI and cognitive decline in an older adults population. Participants with either SVI or OVI had a higher likelihood of experiencing cognitive impairment, even after adjusting for a range of potential confounders, including age, gender, race, education, income, smoking, alcohol use, hypertension, diabetes and stroke. The strength of these associations remained robust across multiple models, underscoring the consistent relationship between visual impairments—both subjective and objective—and cognitive decline.

The robustness of these findings across multiple models and subgroups underscores the strength of the relationship between poor vision and cognitive decline, highlighting the importance of addressing VI in efforts to mitigate cognitive deterioration in the older adults.

### Comparison with previous research and advantages of using NHANES data

Our findings align with previous research that has identified a link between VI and cognitive decline in older adults (15, 16). For instance, studies by reported significant associations between poor vision and cognitive impairment, suggesting that vision loss could be a contributing factor to cognitive decline through mechanisms such as reduced sensory input and increased social isolation (10). Our study supports these conclusions by demonstrating a strong association between VI and limited cognition, even after controlling for a comprehensive range of covariates. However, our study differs from some previous research in its use of the NHANES dataset, which provides a nationally representative sample of the U.S. population. This enhances the generalizability of our findings compared to studies with more localized or specific population samples. Additionally, our study adjusted for a broader range of covariates, including socioeconomic factors such as education level and poverty income ratio (PIR), which may influence both vision and cognitive outcomes. By accounting for these potential confounders, our study provides a more robust analysis of the relationship between VI and cognitive decline. A novel aspect of our study is the detailed subgroup analysis, which examined the association across different demographic and health-related subgroups. This approach allowed us to identify specific groups where the relationship between VI and limited cognition is particularly strong, such as the older adults.

### Biological and psychosocial mechanisms

Several biological and psychosocial mechanisms may explain the observed association between VI and limited cognition. One primary mechanism is reduced sensory input. VI decreases the amount of visual information available to the brain, increasing cognitive load as the brain works harder to process limited and unclear sensory data. This increased cognitive load may accelerate cognitive deterioration, as the brain’s resources are taxed more heavily to compensate for the sensory deficit (17, 18). Reduced physical activity also plays a crucial role in this relationship. VI can limit an individual’s mobility and participation in physical activities, which are important for maintaining cognitive function. Physical activity promotes cardiovascular health and increases blood flow to the brain, supporting cognitive processes and overall brain health. A decline in physical activity due to vision problems can thus contribute to cognitive decline (19). Social isolation is another significant factor linking VI to cognitive decline. VI can hinder an individual’s ability to engage in social activities, leading to reduced social interactions. Studies have shown that social isolation is associated with an increased risk of cognitive decline and dementia. Reduced social engagement limits cognitive stimulation, which is essential for maintaining cognitive health, thereby contributing to cognitive impairment in individuals with poor vision (20). Furthermore, VI may lead to depression and anxiety, which are risk factors for cognitive decline (21). The psychological burden of living with impaired vision can negatively impact mental health, further exacerbating cognitive deterioration. Addressing these interconnected pathways is crucial for understanding how VI influences cognitive health and for developing interventions to mitigate these effects.

### Subgroup analysis and non-linear associations

The subgroup analysis revealed some notable differences in the association between visual impairments and cognitive decline across demographic and health-related factors. The relationship between SVI and cognitive impairment was stronger in males, while OVI showed a more pronounced association in females. This inconsistency between SVI and OVI across gender highlights the potential for different underlying mechanisms driving cognitive decline in men and women with visual impairments. These differences may be influenced by biological, social, and behavioral factors. In males, the stronger association might be due to differences in health-seeking behaviors and social roles, with men possibly delaying treatment for vision problems, thereby exacerbating cognitive effects (22). In terms of age, both SVI and OVI were more strongly associated with cognitive decline in older participants (aged 70–85), suggesting that the impact of visual impairments on cognition may intensify with age. The effect of VI on cognition was more pronounced in alcohol consumers. Studies have shown that light to moderate drinking is associated with a lower risk of cognitive impairment (23). These insights highlight the importance of considering demographic and health-related factors in studying the relationship between SVI and limited cognition, suggesting targeted interventions for specific at-risk groups to effectively prevent cognitive decline related to VI.

We used restricted cubic splines to capture potential non-linear associations between visual impairment and cognitive function. Unlike linear models, this method allows us to explore complex relationships without assuming linearity, providing a more accurate estimation of how visual impairment impacts cognitive function across different score ranges. It also offers an intuitive way to visualize and interpret these non-linear effects. The restricted cubic spline analysis revealed a non-linear relationship between cognitive score and VI, indicating that the impact of VI on cognitive function is not uniform across all levels of cognitive scores. This non-linearity suggests that there may be specific thresholds or ranges of cognitive scores where the detrimental effects of VI are more pronounced. The implications of these non-linear findings are significant. Identifying thresholds where VI has a more substantial impact on cognition can help in pinpointing critical periods for intervention. For instance, individuals within certain cognitive score ranges might benefit more from targeted vision rehabilitation and cognitive training programs. These interventions could be strategically implemented to mitigate the risk of further cognitive decline, especially in those already showing early signs of impairment (14, 24). Future research should aim to identify the precise cognitive score thresholds where VI’s impact is most significant. Such studies could use larger datasets and longitudinal designs to validate and expand upon these findings, providing a more detailed understanding of how VI influences cognitive trajectories over time. From a clinical perspective, understanding this non-linear relationship can guide healthcare providers in developing more personalized care plans. By recognizing the varying impacts of VI across different cognitive levels, interventions can be tailored to the individual needs of patients, improving the effectiveness of treatments aimed at preserving cognitive function in the older adults population (25).

### Clinical and public health implications

The findings of this study have significant clinical and public health implications. Recent research has increasingly recognized VI as a modifiable risk factor for dementia. Livingston et al. showed that untreated vision loss, alongside other risk factors like hypertension, can significantly increase the global burden of dementia, emphasizing the need for early interventions to mitigate this risk (26). Another longitudinal study also demonstrated that self-reported visual impairment was associated with an increased risk of cognitive dysfunction among older adults in China (27).

Regular vision assessments should be incorporated into routine care for the older adults to identify and address VI early. Such proactive measures could help prevent or mitigate cognitive decline associated with VI. Clinically, healthcare providers should prioritize comprehensive eye exams for older adults, integrating these assessments into regular health check-ups. Early detection and intervention for visual impairments, particularly through routine vision screenings, could play a critical role in preventing or slowing cognitive decline in older adults. Incorporating regular vision assessments into standard geriatric care may allow healthcare providers to identify visual impairments before they exacerbate cognitive issues. Early detection of vision problems can lead to timely interventions, such as prescribing corrective lenses, providing low vision aids, or referring patients for surgical treatments like cataract removal. These interventions could help maintain or improve vision, thereby potentially reducing the cognitive load associated with poor vision and slowing cognitive decline (28).

From a public health perspective, several strategies could be implemented to address VI and its broader impacts on health. Public health campaigns can raise awareness about the importance of regular eye exams and educate older adults on the signs and symptoms of vision problems. Community-based programs could provide accessible vision screenings, especially in underserved areas where healthcare resources are limited. Policies that support the integration of vision care into primary healthcare services are also crucial. For example, ensuring that vision care is covered by insurance for the older adults could remove financial barriers to accessing necessary eye care services. Additionally, training healthcare providers to recognize the interplay between VI and cognitive decline can enhance the holistic management of older adults patients. In conclusion, incorporating regular vision assessments into routine older adults care and implementing targeted public health strategies can significantly impact the cognitive health of older adults. By addressing VI early and effectively, we can enhance the quality of life and cognitive function of the aging population, thereby reducing the overall burden on healthcare systems.

### Limitations

There are several important limitations to consider. The cross-sectional design of the study precludes the ability to infer causality between VI and cognitive decline. Longitudinal studies are needed to establish temporal relationships and causative pathways. Another limitation is the potential for residual confounding despite comprehensive covariate adjustment. There may be unmeasured factors influencing both vision and cognition that we did not account for in our analyses. Furthermore, the wide confidence intervals observed in some subgroup analyses, particularly among smokers, highlight the need for larger sample sizes in these specific groups to obtain more precise estimates.

## Conclusion

In conclusion, this study has demonstrated a significant association between visual impairment and limited cognition in the older adults population, even after adjusting for a comprehensive range of covariates. These findings underscore the importance of addressing VI as a crucial component of strategies aimed at preserving cognitive health in older adults. By identifying and treating vision problems early, we can potentially mitigate cognitive decline and improve the overall quality of life for the older adults. Future research should explore the potential of integrating routine vision screenings into standard cognitive health assessments for older adults. Additionally, specific interventions, such as vision rehabilitation programs and the use of corrective lenses, should be tested in clinical trials to determine their effectiveness in preventing or slowing cognitive decline.

## Data Availability

Publicly available datasets were analyzed in this study. This data can be found: https://www.cdc.gov/nchs/nhanes/index.htm.

## Footnotes

^1^https://wwwn.cdc.gov/nchs/nhanes/analyticguidelines.aspx
